# Hypoxia-induced exosomes facilitate lung pre-metastatic niche formation in hepatocellular carcinoma through the miR-4508-RFX1-IL17A-p38 MAPK-NF-κB pathway

**DOI:** 10.7150/ijbs.86767

**Published:** 2023-09-04

**Authors:** Wentao Jia, Shufang Liang, Wanfu Lin, Shu Li, Jiaying Yuan, Mingming Jin, Shuchang Nie, Ya'ni Zhang, Xiaofeng Zhai, Liping Zhou, Changquan Ling, Binbin Cheng, Chen Ling

**Affiliations:** 1 Oncology Department of Traditional Chinese Medicine, the First Affiliated Hospital of Naval Medical University, Shanghai 200433, China.; 2 Faculty of Traditional Chinese Medicine, Naval Medical University, Shanghai, 200043, China.; 3 Department of Gastroenterology, Baoshan Hospital of Integrated Traditional Chinese and Western Medicine, Shanghai University of Traditional Chinese Medicine, Shanghai 201900, China.; 4 Department of Respiratory and Critical Care Medicine, the First Affiliated Hospital of Naval Medical University, Shanghai 200433, China.; 5 Shanghai Key Laboratory of Molecular Imaging, Shanghai University of Medicine and Health Sciences, Shanghai 201318, China.; 6 State Key Laboratory of Genetic Engineering and Engineering Research Center of Gene Technology (Ministry of Education), School of Life Sciences, Zhongshan Hospital, Fudan University, Shanghai, 200438, China.; 7 Department of Clinical Laboratory, The First Affiliated Hospital of Wenzhou Medical University, Wenzhou, Zhejiang, 325000, China.

**Keywords:** Tumor-derived exosomes, Pre-metastatic niche, Hypoxia, MicroRNA

## Abstract

**Background:** Hypoxia plays an important role in the lung metastasis of hepatocellular carcinoma (HCC). However, the process by which hypoxia promotes the formation of a pre-metastatic niche (PMN) and its underlying mechanism remain unclear.

**Methods:** Exosomes derived from normoxic and hypoxic HCC cells were collected to induce fibroblast activation *in vitro* and PMN formation *in vivo.* The micro RNA (miR) profiles of the exosomes were sequenced to identify differentially expressed miRNAs. Gain- and loss-of-function analyses were performed to investigate miR-4508 function. Dual-luciferase, western blotting, and real-time reverse transcription-PCR analyses were used to identify the direct targets of miR-4508 and its downstream signaling pathways. To demonstrate the roles of hypoxic tumor-derived exosomes (H-TDEs) and miR-4508 in the lung metastasis of liver cancer, H22 tumor cells were injected through the tail vein of mice. Blood plasma-derived exosomes from patients with HCC who underwent transarterial chemoembolization (TACE) were applied to determine clinical correlations.

**Results:** We demonstrated that H-TDEs activated lung fibroblasts and facilitated PMN formation, thereby promoting lung metastasis in mice. Screening for upregulated exosomal miRNAs revealed that miR-4508 and its target, regulatory factor X1 (RFX1), were involved in H-TDE-induced lung PMN formation. Moreover, miR-4508 was significantly upregulated in plasma exosomes derived from patients with HCC after TACE. We confirmed that the p38 MAPK-NF-κB signaling pathway is involved in RFX1 knockdown-induced fibroblast activation and PMN formation. In addition, IL17A, a downstream target of RFX1, was identified as a link between RFX1 knockdown and p38 MAPK activation in fibroblasts.

**Conclusion:** Hypoxia enhances the release of TDEs enriched with miR-4508, thereby promoting lung PMN formation by targeting the RFX1-IL17A-p38 MAPK-NF-κB pathway. These findings highlight a novel mechanism underlying hypoxia-induced pulmonary metastasis of HCC.

## Introduction

Hepatocellular carcinoma (HCC), a common malignancy, ranks sixth in incidence and fourth in mortality among all cancer types globally 1. Moreover, HCC is prone to both intrahepatic and extrahepatic metastases. It has been reported that extrahepatic metastasis occurs in 13.5 to 2% of patients with HCC, with a one-year survival rate of less than 25% and a median survival time of only 4.9 to 7 months 2. In patients with lung metastasis, the clinical outcomes even worse. Studies have found that the mortality of patients with lung metastasis within 1 month is 51.65%. In addition, presence of lung metastasis in patients with HCC predicts poor response to sorafenib treatment 3,4.

The lung is the most common site of extrahepatic HCC metastasis, accounting for 34-58% of cases; this represents a major concern in patients with HCC because of limited therapeutic strategies 5-7. Tumor metastasis is a complex process that includes the dissemination of primary tumor cells into the circulatory system, extravasation into secondary organs, and formation of metastatic tumors 8. According to the “seed and soil” theory, primary tumors are capable of developing a favorable microenvironment suitable for tumor cell colonization and growth in the target organs, termed the pre-metastatic niche (PMN). PMNs are characterized by increased vascular permeability, modified extracellular matrix (ECM), recruited bone marrow-derived cells, and immunosuppression in target organs 9. At the initial stage of PMN formation, tumor-derived factors are transported via the blood circulation and serve to increase vascular permeability in the target organs 10. Exosomes, a heterogeneous class of lipid bilayer-enclosed nano-sized vesicles, play an important role in cell-to-cell communication. Tumor-derived exosomes (TDEs) transfer multiple bioactive molecules, including nucleic acids (mRNAs and non-coding RNAs) and proteins, to metastatic sites, contributing to PMN establishment. For example, TDEs transfer miR-3473b to lung fibroblasts to promote lung tumor cell intrapulmonary colonization by activating the NF-κB pathway 11.

Hypoxia is a common characteristic of the solid tumor microenvironment, especially in HCC. In addition, several therapies such as incomplete ablation and transarterial chemoembolization (TACE) may aggravate local hypoxia, thereby promoting tumor progression and metastasis 12-14. Specifically, a 3.2-fold risk for pulmonary metastasis following TACE has been reported in patients with HCC 13. The median interval between initial diagnosis and pulmonary metastasis was lower in patients with HCC underwent TACE, the time reduced about 29% compared to patients without TACE 13. Recently, studies have established that elevated S100A9 induced by TACE in HCC tissues can promote invasion of cancer cells through PGAM5-ROS pathway and further facilitate progression and metastasis 15. Recent studies have emphasized that hypoxia not only directly promotes the invasion and stemness of tumor cells^16^ but also drives cancer cells to produce more cytokines, non-coding RNAs, and exosomes, which are involved in ECM modification, vascular permeability enhancement, and pro-inflammatory regulation 17. For example, proteins, including VASP and YTHDF1, and non-coding RNAs, such as circ-LNPEP and miR-92a, exert tumorigenic effects through multiple pathways involving the direct regulation of tumor cells and ECM remodeling 18-21.

In the present study, we propose a mechanism underlying the increased risk of lung metastasis caused by regional hypoxia in patients with HCC. We found that hypoxia induced the upregulation of miR-4508 in TDEs and that this miRNA could bind to the 3′UTR of regulatory factor X1 (RFX1). Downregulation of RFX1 activates fibroblasts through the IL17A-p38 MAPK-NF-κB signaling pathway and promotes PMN formation. Activated fibroblasts promote the differentiation and recruitment of myeloid-derived suppressor cells (MDSCs). In turn, pulmonary PMNs established by activated fibroblasts facilitate lung metastasis of primary tumors *in vivo*. Our results suggest a new miRNA cargo in exosomes as well as its target as important factors for fibroblast activation and PMN formation in HCC. Moreover, we introduce a novel mechanism to explain the risk of TACE in inducing lung metastasis. Together, our findings highlight the role of hypoxic TDEs (H-TDEs) in distant PMN formation.

## Methods

### Reagents and antibodies

Antibodies against TSG101 (#28283-1-AP, 1:500), CD63 (#25682-1-AP, 1:500), and RFX1 (#26859-1-AP, 1:1000) were purchased from Proteintech (Wuhan, China). Antibodies against periostin (POSTN; #91771; 1:1000), fibroblast-activating protein (FAP; #66562; 1:1000), α-smooth muscle actin (α-SMA; #19245; 1:1000), fibronectin (FN; #26836; 1:1000), GAPDH (#5174; 1:1000), p38 MAPK (#8690; 1:1000), phosphorylated p38 (p-p38) MAPK (#4511; 1:1000), IκBα (#9247; 1:1000), NF-κB p65 (#8242; 1:1000), and histone H3 (#4499; 1:1000) were purchased from Cell Signaling Technology (Boston, MA, USA). Murine anti-RFX1 antibody (sc-374270; 1:500) was purchased from Santa Cruz Biotechnology (Dallas, TX, USA). Antibodies for flow cytometry, including APC-labelled anti-CD11b (#101212), PE-labelled anti-GR1 (#108408), and APC-labelled anti-human CD326 (EpCAM; #369809) were purchased from BioLegend (San Jose, CA, USA). FITC-labelled anti-human CD90 (Thy-1; #03011) was purchased from PeproTech (Boston, MA, USA). APC-labelled IgG2b (#400611, BioLegend) and PE-labelled IgG2b (#400607, BioLegend) were used as isotype controls. FITC-labelled anti-human CD90 (#03011-50) was purchased from PeproTech (Boston, MA, USA). The cellular exosome inhibitor GW4869 (M4974) was purchased from AbMole (Shanghai, China). The NF-κB signaling inhibitor (-)-DHMEQ (HY-14645) and p38 MAPK inhibitor (HY-10256) were purchased from MedChemExpress (Monmouth Junction, NJ, USA). RNA oligonucleotides (miR mimics, miR inhibitors, and siRNAs) were designed and synthesized by GenePharma (Shanghai, China).

### Cell culture

Human HCC cell lines HCCLM3, MHCC-97H, and Huh-7, human embryonic lung fibroblast MRC-5, and murine liver cancer cell line Hepa1-6 were purchased from the Cell Bank of the Chinese Academy of Science (Shanghai, China) and cultured in Dulbecco's modified Eagle's medium (DMEM; Gibco, Erie, NY, USA) supplemented with 10% fetal bovine serum (FBS; 10099141c; Gibco). The murine liver cancer cell line luciferase-labeled H22 (H22-Luc) was obtained from Shanghai Fuheng Biotechnology Co., Ltd. (Shanghai, China) and cultured in complete DMEM. The murine lung fibroblast WML2 cell line was purchased from Guangzhou Jennio Biotech Co., Ltd. (Guangzhou, Guangdong, China). Cells were incubated at 37 °C in 5% CO_2_-95% air atmosphere. For hypoxic treatment, the cells were cultured in a 3-gas hypoxia incubator with 1% O_2_, 5% CO_2_, and 94% N_2_ for the indicated time periods (Binder, Tuttlington, Germany).

### Preparation of the conditioned medium

HCC cells were maintained in DMEM supplemented with 10% FBS without exosomes (OriCell, Guangdong, China). The cells were cultured under normoxic or hypoxic conditions for 24 h. The conditioned medium (CM) was harvested and centrifuged at 4000 × *g* for 15 min to remove cellular debris. The cleaned CM was stored at -80 °C for future use.

To obtain activated fibroblasts, CM, MRC5, and WML2 cells were seeded in 6-well plates and pretreated (for 48 h) with exosomes derived from HCC cells transfected with or without miRNA-4508 mimic. The cells were then washed twice with PBS and cultured in 2 mL of complete DMEM for another 24 h. The CM was collected for future use.

### Isolation and identification of exosomes from cell cultures and blood plasma

Exosomes from CM were isolated using the density-gradient ultracentrifugation method as described previously 22. Briefly, the supernatant was centrifuged at 10,000 × *g* at 4 °C for 45 min to remove large sloughy vesicles. Then, the supernatant was filtered through a 0.45 μm filter and centrifuged at 100,000 × *g*, 4 °C for 70 min. Exosome pellets were re-suspended in PBS and subjected to another ultracentrifugation at 100,000 × *g*, 4 °C for 1 h to wash the exosomes. The washed exosomes were thoroughly resuspended in PBS and stored at -80 °C for future use.

To isolate exosomes from patient plasma, 5 mL of venous blood was collected from fasting patients with HCC the morning prior to TACE and on days 3 and 5 following TACE. The harvested blood was centrifuged at 3000 rpm, 4 °C for 15 min and the obtained plasma collected and stored at -80 °C. Plasma exosomes were isolated from 2 mL of plasma using an ExoEasy Maxi kit (Qiagen, Hilden, Germany), following the manufacturer's instructions. Briefly, the pre-filtered plasma was mixed with an equal volume of XBP buffer. The exosomes were incubated, absorbed onto the membrane of an exoEasy spin column, and washed twice with XWP buffer. Total exosomes were then diluted in 400 μL of XE buffer. The use for this study of patient plasma was approved by the Ethics Committee of Changhai Hospital.

Exosome morphology and size were observed using a transmission electron microscope (Hitachi, Tokyo, Japan). Nanoparticle tracking analysis (NTA) was performed using a ZetaVIEW (Particle Metrix, Ammersee, Germany). Exosomes were also identified by determining the expression of the specific markers, TSG101 and CD63.

### Exosome tracing

For *in vitro* tracing, 40 μg of TDEs were labeled with PKH26 (UR53202; UmiBio, Shanghai, China) following the manufacturer's instructions. Briefly, PKH26 dye was mixed with diluent C and incubated with the exosomes at room temperature for 10 min. The free dye and nonspecifically stained micellular structures were then removed through density-gradient ultracentrifugation. Labelled exosomes were incubated with MRC5 cells for 24 h. Images of exosome uptake were captured using a confocal microscope (SP8; Leica, Wetzlar, Germany).

For *in vivo* tracing, TDEs were labeled with DIR (UR21017; Umibio) following the manufacturer's instructions. Next, 40 μg DIR-labeled exosomes were intravenously injected into BALB/c mice. Exosome uptake in specific organs was observed using a live fluorescence imaging system (Caliper Life Sciences, Boston, MA, USA) at time points of 30 min, 2 h, and 4 h following injection. The exosome-treated mice were sacrificed. Lung tissues were frozen and stained with DAPI (Abcam, Cambridge, UK). Images were captured using a fluorescence microscope (SP8; Leica). All experiments were approved by the Committee on the Ethics of Animal Experiments of Naval Medical University.

### Migration assay

A total of 8 × 10^4^ TDE-treated MRC5 cells were seeded in the upper chamber of a transwell system (Corning Costar Corp., Armonk, NY, USA). DMEM containing 10% FBS was added to the lower chamber. After 24 h, migrated cells were fixed with 4% polymethanol and stained with 1% crystal violet. Images were captured using a light microscope (SP8; Leica). All experiments were performed in triplicate and repeated three times.

### Western blotting

Cells or exosomes were lysed in Cell Lysis Buffer (#9803; CST, Danvers, MA, USA) supplemented with 1 mM PMSF and a protease/phosphatase inhibitor cocktail (KangChen, Shanghai, China). Total protein was quantified using a BCA assay kit (Thermo Fisher Scientific, Waltham, MA, USA) and denatured at 95 °C for 10 min in loading buffer (Yeasen Biotechnology, Shanghai, China). Equal amounts (20 μg) of protein were loaded onto SDS-PAGE gels and transferred onto PVDF membranes (Millipore, Billerica, MA, USA). Membranes were blocked in 5% skim milk for 2 h and incubated for 12 h at 4 °C with primary antibodies. After incubation with horseradish peroxidase-conjugated secondary antibodies for 1 h at room temperature, the bands were visualized using chemiluminescence with SuperSignal substrate (#34096; Thermo Fisher Scientific). Autoluminography was performed using a G:BOX Gel imager (SYNGENE, Cambridge, UK). The antibodies used for western blotting analysis are listed in the “Reagents and antibodies” section above.

### Real time RT-PCR

Total RNA was extracted using TRIzol reagent (Invitrogen, Carlsbad, CA, USA); miRNAs from the exosomes were extracted using the miRNeasy Micro Kit (#217084, Qiagen). Total RNA concentration was determined using a NanoDrop One (Thermo Fisher Scientific). A total of 500 ng of RNA and the same volume of miRNA (7 μL) were used for cDNA synthesis using PrimeScript RT reagent (TaKaRa, Otsu, Shiga, Japan) following the manufacturer's instructions. Real-time PCR was performed using the SYBR Premix (ToYoBo, Osaka, Japan) with a StepOnePlus Real-Time PCR instrument (Applied Biosystems, Foster City, CA, USA). *Caenorhabditis elegans*-miR-39 was used as an external reference for the normalization of exosomal miRNAs. The relative expression was calculated using the 2^-△△Ct^ method.

### Animal models

Male BALB/c mice (6-8 weeks old, 20 ± 2 g) were purchased from SLAC Laboratory Animal Co., Ltd. and maintained on a 12:12 h light/dark cycle at the Laboratory Animal Center of Naval Medical University. The mice were randomly divided into three groups (n = 8 each). Pulmonary PMN models were established via the intravenous injection of exosomes from Hepa1-6 cells cultured under normoxic or hypoxic conditions. The control animals were injected with equal volumes of PBS. Exosomes were injected three times per week for 3 weeks. Two mice from each group were euthanized for PMN determination. Experimental lung metastases were induced by injecting H22-Luc cells into the tail vein (4 × 10^6^ cells in 200 mL of PBS). On days 7, 14, and 21, lung metastases were determined based on images captured using a live animal fluorescence imaging system (Caliper Life Sciences).

### Immunofluorescence staining

Immunofluorescence (IF) staining of mouse lung sections was performed according to standard protocols. Paraffin-embedded sections were deparaffinized, rehydrated, and subjected to antigen retrieval. The slides were then incubated with primary antibodies (1:200 to 1:500) at 4 °C overnight. After washing three times with PBS, the slides were stained with Alexa Fluor 488 (1:2000) or Alexa Fluor 594 (1:1000) (Life Technologies, Carlsbad, CA, USA) for 1 h at room temperature. Nuclei were stained with DAPI. Images were captured using a fluorescence microscope (Leica).

### Immunohistochemistry

Immunohistochemistry (IHC) of mouse lung sections was performed according to standard protocols. Paraffin-embedded sections were deparaffinized, rehydrated, and subjected to antigen retrieval. The slides were then incubated with primary antibodies (1:200 to 1:500) at 4 °C overnight. After washing three times with PBS, the slides were stained with secondary antibodies (Abcam) for 1 h at room temperature. DAB (Thermo Fisher Scientific) was used for chromogenic detection. Histological sections were observed under a bright-field microscope, and representative fields were photographed at ×10 or ×20 magnification.

### Hematoxylin and eosin staining

Pulmonary metastatic lesions were determined using hematoxylin and eosin (H&E) staining according to standard protocols. Briefly, paraffin-embedded sections were deparaffinized, rehydrated, and stained with H&E. Sections were then dehydrated using an ethanol gradient and cleared in xylene. Images were captured using an inverted microscope (Leica).

### miRNA sequencing

Exosomal RNA was extracted from the CM of HCCLM3 and MHCC-97H cells cultured in hypoxic and normoxic conditions using TRIzol reagent (Takara, Japan). Libraries for miRNA sequencing were prepared using RT-PCR. miRNA sequencing was performed using an Illumina HiSeq 2500 (San Diego, CA, USA). Differentially expressed genes were analyzed using the DESeq2 package based on the empirical Bayes method. Significant differential expression of miRNAs was defined as a fold change ≥ 2 and p-value < 0.05.

### Cell transient transfection

Cells were seeded in 6-well plates or 10 cm dishes at 70% confluence prior to transfection. Transient transfections of plasmids and miRNA mimics were performed using Attractene Transfection Reagent (301005, Qiagen) following the manufacturer's instructions.

### Dual-luciferase reporter assay

The 3′UTR region of the *RFX1* gene containing a miR-4508 binding site, as well as mutant sequence were synthesized and cloned in the GV716 vector. Wide-type/mutant *RFX1* (3′UTR)-firefly luciferase plasmids were co-transfected with miR-4508 mimics or control miRNA into 293T cells using the Attractene Transfection Reagent (301005, Qiagen) according to the manufacturer's protocol. A *Renilla* luciferase-expressing plasmid was used as an internal reference. After 48 h, the cells were lysed and luciferase activity was determined using a dual-luciferase reporter assay system (Promega, Madison, WI, USA). The assay was performed in triplicate and repeated three times.

### Transmission of CY3-labelled miRNA

For the miR-CY3 transfer assay, HCCLM3 cells transfected with CY3-labelled miR-4508 (LM3-miR-CY3) were seeded into the upper chamber of the transwell co-culture system, and MRC5 cells were seeded into the lower chamber. In the control group, miR-CY3 was added directly to the upper chamber. After 24 h, CY3 fluorescence in MRC5 cells was measured to determine the miRNA transmission ability of the HCCLM3-derived exosomes.

### Bioinformatics analysis

Bioinformatics tools, including miRDB, miRTarBase, miRWalk, and TargetScan, were used to predict the potential targets of miR-4508. Candidate mRNA 3′UTRs retrieved in two or more databases were selected. Gene ontology (GO) enrichment analysis of the biological process (BP) category was performed using the DAVID bioinformatics tool. For analyzing the expression of *RFX1* in tumor and normal tissues, RNA-sequencing expression (level 3) profiles and corresponding clinical information for patients with metastatic lung tumors were downloaded from the TCGA database (https://portal.gdc.cancer.gov/). To calculate single-sample gene set enrichment, the R software GSVA package was used to derive the absolute enrichment scores of gene sets from previously validated gene signatures from MsigDB. Differentially enriched gene sets were used to analyze the correlation between *RFX1* expression and pathway scores using Spearman's correlation.

### Statistical analysis

All data are expressed as means ± standard deviation. Student's t-test or one-way analysis of variance was performed to determine the significance of differences between the groups, followed by Tukey's test using SPSS 20.0 (IBM, Armonk, NY, USA). Statistical significance was set at *P* < 0.05.

## Results

### Hypoxic HCC cell-derived exosomes promote fibroblast activation

Hypoxia within the primary tumor leads to the secretion of multiple previously identified and as-yet-unidentified tumor-derived factors that contribute to PMN formation 23. Lung fibroblasts are the main source of inflammatory cytokines and ECM. Therefore, we examined the effects of hypoxia on the ability of HCC cells to promote fibroblast activation* in vitro*. CM from several HCC cell lines cultured under normoxic or hypoxic conditions was collected and used to treat MRC5 cells. We found that CM from hypoxic HCC cell-treated fibroblasts led to the production of more α-SMA, FAP, and POSTN (Fig. S1A). However, the survival of MRC5 cells remained unaltered (Fig. S1B).

**Figure 1:**
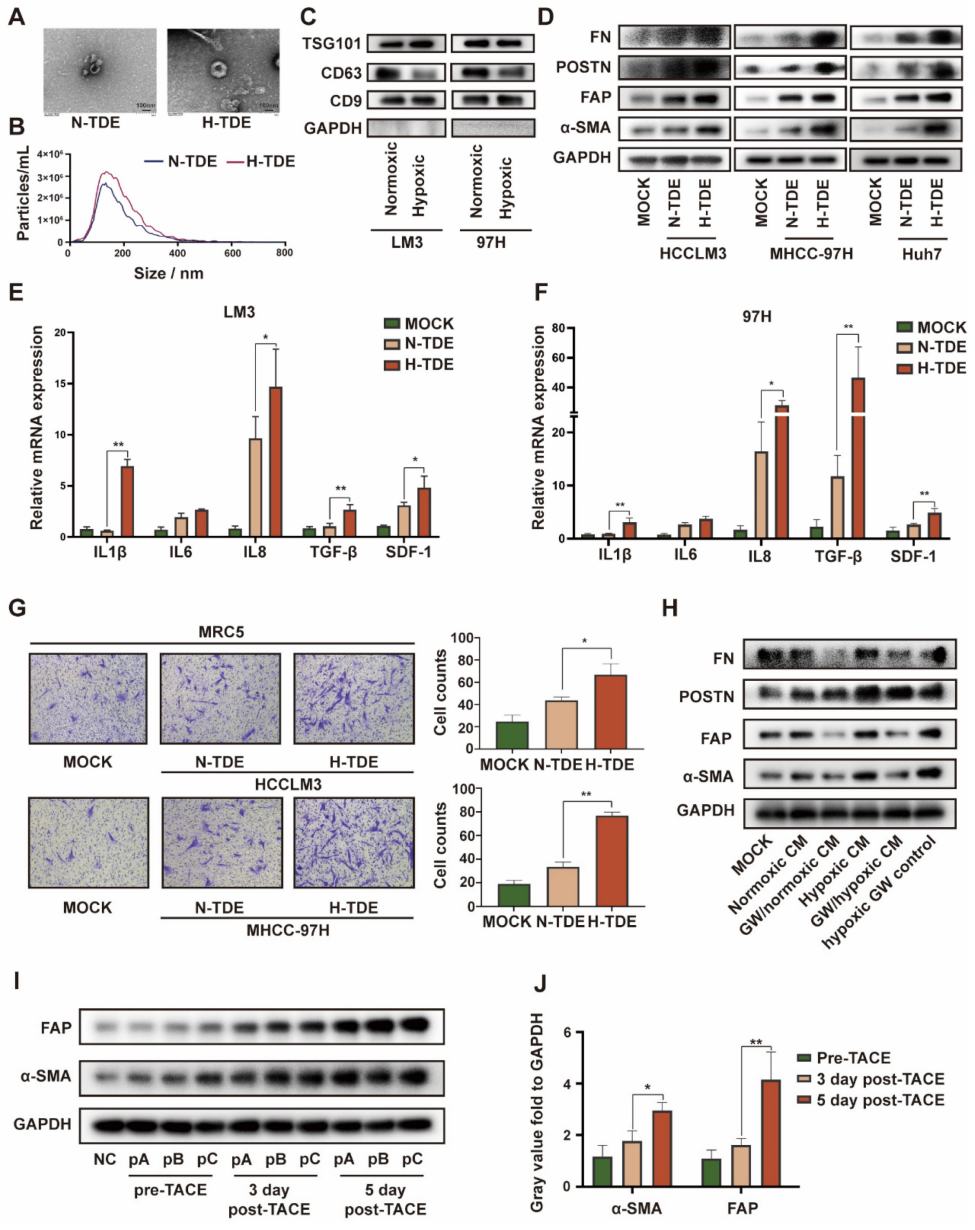
H-TDEs promote the activation of fibroblasts* in vitro.* (A) Transmission electron micrographs of TDEs. (B) Size of TDEs as determined by NTA. (C) Expression of characteristic exosomal markers TSG101, CD9, and CD63 as measured by WB; GAPDH band was present as a negative marker for TDEs. (D) Expression of fibroblast activation markers in MRC5 cells treated with equal quantities of N-TDEs or H-TDEs. (E) and (F) Relative expression of pro-inflammatory factor mRNA in MRC5 cells treated with equal quantities of N-TDEs or H-TDEs from HCCLM3 and MHCC-97H cells. (G) Migration of MRC5 cells treated with equal quantities of exosomes.; (H) Effect of GW4869 (GW) on the expression of fibroblast activation markers induced by CM of HCCLM3 cells. (I) Effect of plasma-derived exosomes from patients with HCC before and after TACE on the expression of fibroblast activation markers in MRC5 cells (NC: MRC5 cells cultured without plasma-derived exosomes; pA, pB, pC: MRC5 cells treated with plasma-derived exosomes from three patients). (J) Quantified gray value of each band presented in Fig. 1I; each bar represents the mean ± S.D.; each experiment was repeated at least 3 times; *P < 0.05, **P < 0.01.

Because exosomes play a key role in cellular communication, we isolated and purified exosomes from HCCLM3 cells cultured under normoxic and hypoxic conditions. Transmission electron micrographs revealed the typical “cup-plate” shape of exosomes (Fig. 1A). Notably, NTA indicated that the sizes of the exosomes secreted by HCC cells cultured under different conditions were comparable (Fig. 1B), whereas a higher quantity of exosomes was secreted by hypoxic than normoxic HCC cells (Fig. S1C). Then, the exosomal characteristic markers TSG101, CD9 and CD63 were confirmed by WB (Fig. 1C). CD63, also known as lysosome-associated membrane glycoprotein 3 which indicate lysosomal functions, was downregulated in H-TDEs, suggesting that the higher level of H-TDE secretion might be attributed to lysosomal dysfunction in hypoxic HCC cells 24,25.

To determine the efficiency of exosome intake by MRC5 cells, TDEs were labelled with PKH26 (40 μg in each groups). After incubation with MRC5 for 24 h, PKH26 spots in the MRC5 cells were detected using confocal microscopy (Fig. S2A). To further verify the *in vivo* delivery of exosomes, DIR-labeled exosomes were intravenously injected into the tail vein of mice. The *in vivo* distribution of the TDEs was monitored using a live animal imaging system (Fig. S2B), which revealed that the exosomes were mainly distributed in the lungs and liver. After 4 h, the liver and lungs were removed and examined via fluorescence imaging. The ROI value in the lung reached 5.9 × 10^8^ radiant efficiencies, whereas that of the liver reached 3.1 × 10^9^ radiant efficiencies. Images of frozen lung sections confirmed the trend (Fig. S2C).

We next examined whether H-TDEs could activate fibroblasts to a greater extent than the effect afforded by normoxic TDEs (N-TDEs). The activation markers of fibroblasts, including-SMA, FAP, POSTN, and FN, were markedly upregulated in H-TDE-treated MRC5 cells compared to those in N-TDE-treated MRC5 cells (Fig. 1D). Additionally, the mRNA levels of pro-inflammatory factors and cytokines including IL1β, IL6, IL8, TGF-β, and SDF-1 were significantly increased in H-TDE-treated MRC5 cells compared with those of N-TDE-treated MRC5 cells (Fig. 1E and 1F). Furthermore, compared to that following N-TDE treatment, the migration ability of MRC5 cells was significantly enhanced after treatment with H-TDEs (Fig. 1G). Conversely, the activation of MRC5 cells treated with hypoxic or normoxic HCC-CM was markedly attenuated upon pretreatment with the exosome-release inhibitor GW4869, which did not directly affect MRC5 cell activation (Fig. S2D and 1H). Taken together, these results suggested that hypoxia enhanced HCC cell-induced fibroblast activation through exosome-mediated cellular communication.

To confirm the *in vitro* results, exosomes were collected from the plasma of three patients with HCC who underwent TACE. The levels of α-SMA and FAP in fibroblasts treated with exosomes obtained following TACE were significantly elevated compared with those generated by exosomes obtained prior to TACE (Fig. 1I and IJ). These results indicated that TACE, which may induce a local hypoxic microenvironment, leads to the activation of distant fibroblasts through changes in TDE cargo.

### H-TDEs promote PMN formation and lung metastasis

**Figure 2:**
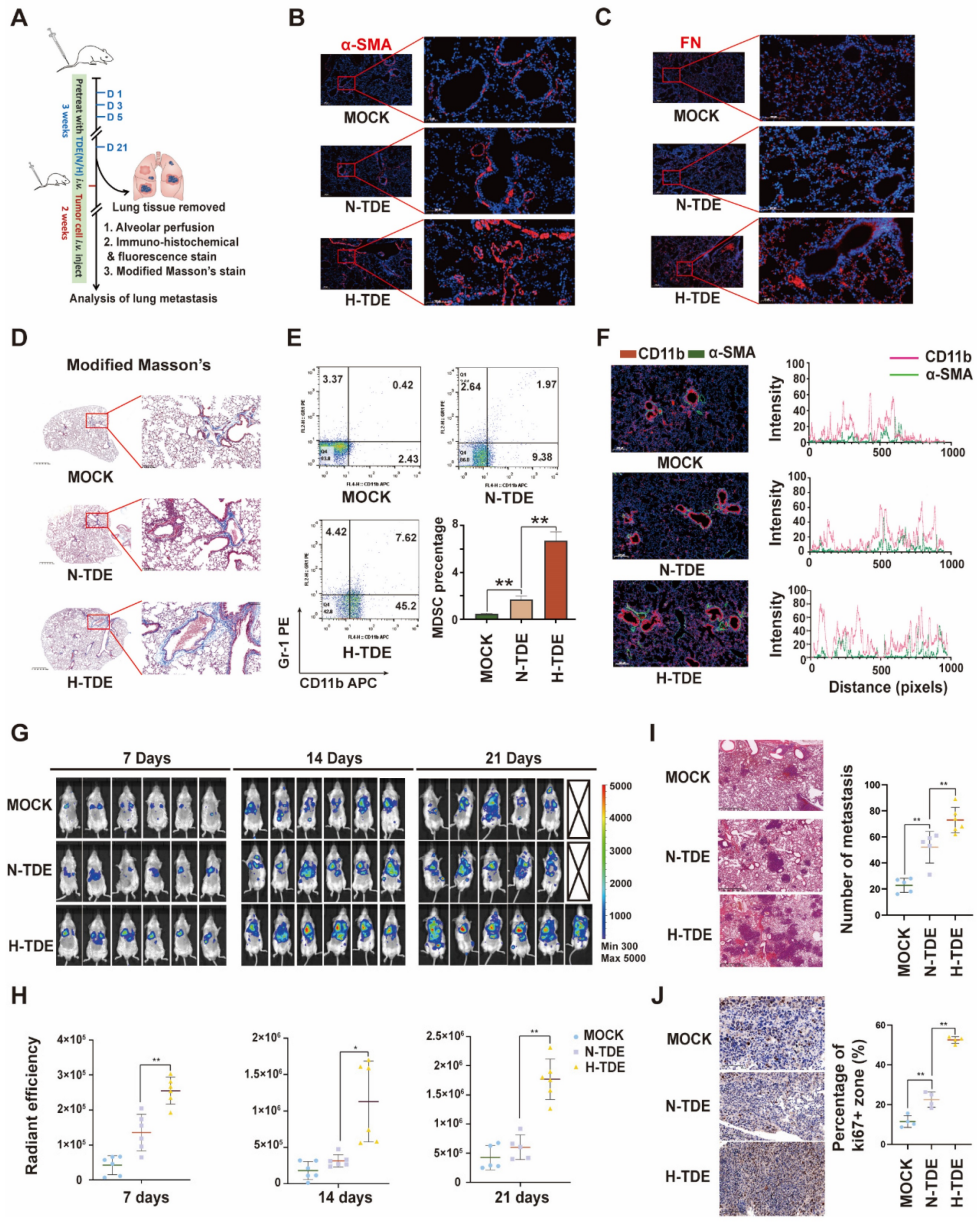
H-TDEs promote PMN formation and lung metastasis. (A) Schematic diagram of the study design. (B) and (C) IF staining of α-SMA and FN in the lungs of the different treatment groups. (D) Modified Masson's staining of the lungs. (E) Percent of CD11b+Gr1+ MDSCs in the lung alveolar perfusion fluid of mice. (F) Double IF staining of CD11b and α-SMA in mouse lungs. (G) IVIS images of mice injected with H22-Luc cells. (H) Quantification of the fluorescence intensity of tumors. (I) and (J) HE staining and Ki67-IHC staining of lungs with metastatic tumors. Each bar represents the mean ± S.D. *P < 0.05, **P < 0.01.

To investigate whether H-TDEs could promote the formation of pulmonary PMNs *in vivo*, N-TDEs or H-TDEs from murine Hepa1-6 HCC cells were injected into BALB/c mice through the tail vein every 3 days for a total of 21 days. The lungs were removed from a subset of animals to evaluate PMN formation. The remaining mice were then injected with H22-Luc tumor cells to create a tumor metastasis model (Fig. 2A). IF staining showed that the α-SMA (Fig. 2B), FN (Fig. 2C) and FAP (Fig. S2E) levels in lung tissues were significantly upregulated following H-TDE treatment. Compared with that of N-TDE-treated animals, modified Masson's staining demonstrated that H-TDEs induced the most severe collagen deposition, suggesting a fibroblast-activating effect (Fig. 2D).

Evaluation of the levels of CD11b+Gr1+ MDSCs, which function as immunosuppressive components in the pulmonary interstitium, in alveolar perfusion liquid revealed that H-TDEs significantly increased the ratio of CD11b+Gr1+ MDSCs compared to that following N-TDE treatment (Fig. 2E). IF co-staining revealed an increase in the co-localization of CD11b+ monocytes (red) and-SMA + fibroblasts (green) in the lungs of H-TDE-treated mice (Fig. 2F), implying that the activation of fibroblasts promotes CD11b+ monocyte infiltration following H-TDE treatment. To determine the role of activated fibroblasts in MDSC differentiation, we cultured primary mouse monocytes *in vitro* in CM collected from TDE-treated murine WML2 pulmonary fibroblast cells. WML2 cells treated with H-TDE exhibited significantly upregulated proportions of CD11b+ GR-1+ MDSCs among mouse monocytes (Fig. S3A), in addition to elevated immunosuppressive factor expression (Fig. S3B). The transition of quiescent tumor cells into proliferative tumor cells which is assisted by activated fibroblasts plays a key role in metastatic outgrowth. We next investigate whether TDE-educated lung fibroblasts have capability enhance aggressiveness of cocultured LM3 tumor cells. We confirmed that tumor stemness markers including SOX2, OCT4 and NANOG can be significantly upregulated by treatment of CM from H-TDE-stimulated MRC5 cells (Fig. S3C).

To investigate whether H-TDEs mediated PMNs are capable promoting metastatic tumor development, metastatic tumors in mice were visualized weekly using IVIS. The results showed that pulmonary metastatic tumors developed more rapidly in H-TDE-pretreated mice than in the other groups, as early as seven days following tumor cell injection (Fig. 2G and 2H). HE staining of lung tissue harvested three weeks after tumor cell injection revealed more metastatic tumors in H-TDE-pretreated mice than in N-TDE-pretreated mice (Fig. 2I); Number of metastasis was measured by analyzing tumor niche > 0.5 mm from H&E-stained slides with maximum cross-section of paraffin-embedded lungs and representative panoramic pictures were present in Fig. S3D. IHC staining (Fig. 2J) showed that Ki67-positive staining was also significantly increased in H-TDE-pretreated mice.

### Upregulated miR-4508 in H-TDEs mediates fibroblast activation

**Figure 3:**
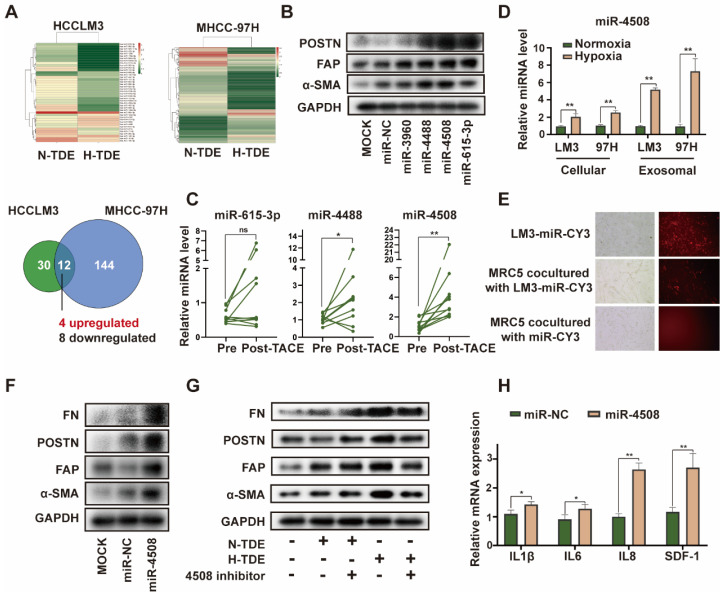
Upregulated miR-4508 in H-TDEs mediates fibroblast activation. (A) High-throughput sequencing of exosomal miRNA from HCCLM3 and MHCC-97H cells. (B) Effect of transferred miR mimics on fibroblast activation. (C) MiR-4508, miR-4488, and miR615-3p levels in plasma-derived exosomes of patients with HCC before and after TACE. (D) MiR-4508 expression in TDEs and tumor cells under hypoxic or normoxic conditions. (E) Intake of HCCLM3-derived exosomal miRNA by MRC5 cells. (F) Effect of miR-4508 mimics on the expression of fibroblast activation markers. (G) Effect of an miR-4508 inhibitor on H-TDE-induced fibroblast activation. (H) Relative expression of inflammation-related mRNA in MRC5 cells transfected with miR-4508 mimics. Each bar represents the mean ± S.D; each experiment was repeated at least 3 times; *P < 0.05, **P < 0.01.

miRNAs are an abundant non-coding RNA in exosomes and play an important role in cell-cell communication 26. Therefore, we hypothesized that miRNAs encapsulated in H-TDEs mediate fibroblast activation. The miRNA profiles of H-TDEs in HCCLM3 and MHCC-97H cells were determined using high-throughput miRNA sequencing (Fig. 3A). Differentially expressed miRNAs were identified as having p < 0.05 and a fold change > 2 between hypoxic state changes in the two cell lines. Four upregulated and eight downregulated miRNAs were identified. Among the differentially expressed miRNAs, upregulated miRNAs (miR-3960, 4488, 4508, and 615-3p) were further investigated. Mimics of miR-4488, miR-4508, and miR-615-3p, but not miR-3960, activated MRC5 to different degrees, with the miR-4508 mimic exhibiting the greatest potential for fibroblast activation as determined by POSTN, FAP, and-SMA expression (Fig. 3B). In plasma-derived exosomes from patients with HCC who underwent TACE, the level of miR-4508 was most significantly upregulated post-TACE compared to pre-TACE levels, with the greatest fold change (Fig. 3C). Evaluation of miR-4508 in HCC cell lines and TDEs showed that both cellular and exosomal miR-4508 levels in HCCLM3 and MHCC-97H cells were significantly upregulated under hypoxic conditions (Fig. 3D). Therefore, we focused on miR-4508 in further investigations.

To confirm whether miR-4508 was transferred into MRC5 cells by TDEs, TDEs were isolated from HCCLM3 tumor cells transfected with CY3-labelled miR-4508. After 24 h, CY3 fluorescence was observed in the MRC5 cells co-cultured with TDEs (Fig. 3E). However, MRC5 cells cultured with miR-CY3 mimics exhibited little CY3 fluorescence. Furthermore, the promoting effect of H-TDEs on MRC5 cell activation (Fig. 3F) was offset by a miR-4508 inhibitor (Fig. 3G). The mRNA levels of pro-inflammatory cytokines including IL-1β, IL6, IL8, and SDF-1 in MRC5 cells were significantly upregulated by miR-4508 mimic transfection (Fig. 3H), suggesting increased pro-inflammatory cytokine secretion. The miR-4508 mimic also increased the migration ability of fibroblasts (Fig. S3E).

### MiR-4508 stimulates fibroblast activation by targeting RFX1

**Figure 4:**
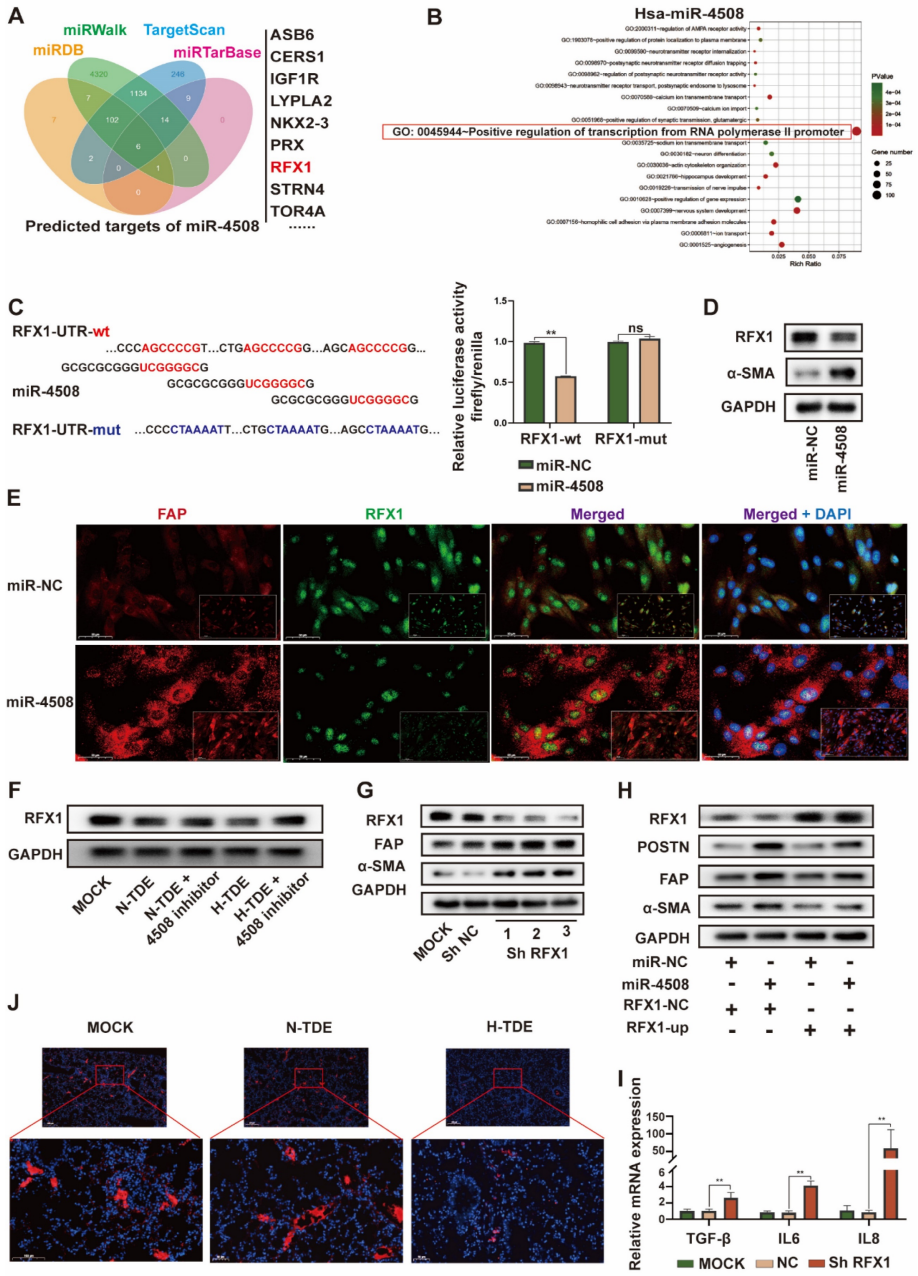
MiR-4508 stimulates fibroblast activation by targeting RFX1. (A) Target gene prediction of miR-4508 using bioinformatics tools. (B) GO analysis of the predicted genes. (C) Wild-type and mutated form of the binding site between miR-4508 and RFX (left); relative luciferase activity of RFX1-wt and RFX1-mut plasmid-transfected cells (right). (D) Expression of RFX1 and α-SMA in MRC5 cells transfected with miR-4508 mimics. (E) IF staining of FAP and RFX1 in MRC5 cells. (F) Effect of an miR-4508 inhibitor on H-TDE-induced downregulation of RFX1 in MRC5 cells. (G) Effect of RFX1 shRNAs on the expression of RFX1, FAP, and α-SMA in MRC5 cells. (H) Effect of an RFX1 overexpression plasmid on miR-4508 mimic-induced downregulation of RFX1 and upregulation of POSTN, FAP, and α-SMA in MRC5 cells. (I) Relative expression of inflammation-related mRNA in MRC5 cells transfected with RFX1 shRNA. (J) IF staining of RFX1 in the lungs of mice treated with TDEs. Each bar represents the mean ± S.D.; each experiment was repeated at least 3 times; *P < 0.05, **P < 0.01.

To further explore the targets of exosomal miR-4508 in fibroblasts, four bioinformatics tools: miRDB, miRTarBase, miRWalk, and TargetScan were applied to predict the target genes. GO analysis of the retrieved candidate targets (Fig. 4A) indicated that “positive regulation of transcription from RNA polymerase II promoter” was the most enriched function of potential miR-4508 targets (Fig. 4B). Notably, this represented the major function of RFX1. Previous studies have described both the tumor-suppressive and oncogenic roles of RFX1, which involve the regulation of proliferation, the immune system, stemness, and chemoresistance 27,28. To confirm that RFX1 is a binding target of miR-4508, a dual-luciferase assay was performed. MiR-4508 transfection decreased the luciferase activity of wild-type RFX1 but not that of its mutant form, indicating the direct binding of miR-4508 mimics to the wild-type *RFX1*-3′UTR (Fig. 4C). Mimics of miR-4508 also led to a reduction in RFX1 protein and upregulation of α-SMA in MRC5 cells (Fig. 4D). The reduction in RFX1 and elevation of FAP in MRC5 cells following miR-4508 transfection were confirmed by IF staining (Fig. 4E). These results suggested that *RFX1* is a target gene of miR-4508. Furthermore, H-TDEs suppressed RFX1 expression in fibroblasts (Fig. 4F), which was abrogated by the miR-4508 inhibitor (Fig. 4F).

Moreover, to determine the function of RFX1 in fibroblast activation, we constructed three shRNA vectors of RFX1 and determined their efficacy by WB (Fig. 4G). RFX1 knockdown increased the expression of FAP and-SMA in fibroblasts (Fig. 4G), whereas RFX1 overexpression neutralized the effects of miR-4508 on the expression of-SMA, FAP, and POSTN in MRC5 cells (Fig. 4H). RFX1 knockdown also elevated the mRNA levels of TGF-β, IL6, and IL8 in MRC5 cells (Fig. 4I). In the lungs of H-TDE-treated mice, the expression of RFX1 was also significantly decreased compared with that in the N-TDE and control groups (Fig. 4J). These results imply that the miR-4508-induced activation of MRC5 cells is mediated by RFX1 downregulation.

### MiR-4508 facilitates the formation of PMN and lung metastasis of HCC

**Figure 5:**
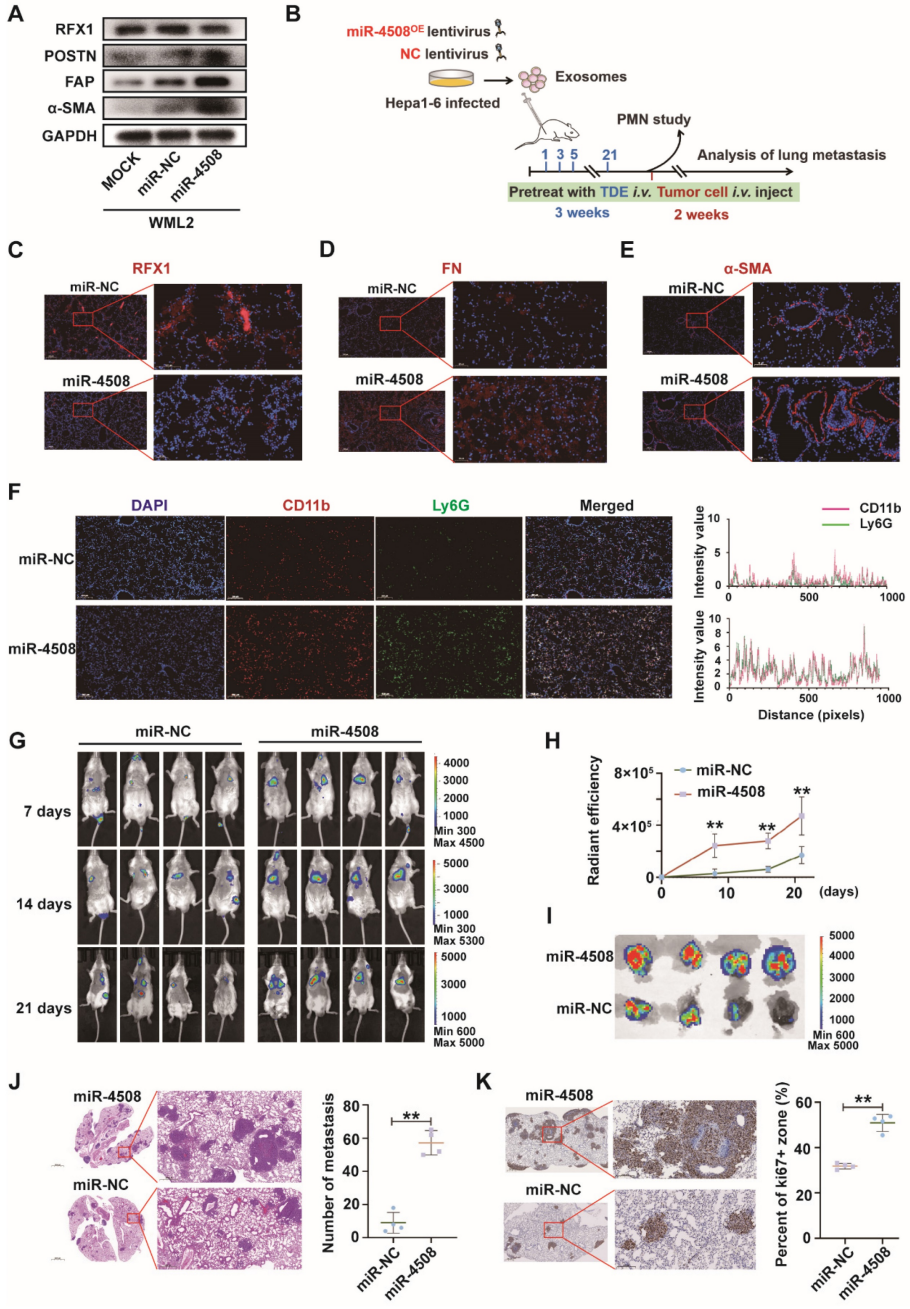
MiR-4508 facilitates the formation of PMN and lung metastasis of HCC. (A) Effect of miR-4508 on mouse lung fibroblasts. (B) Schematic diagram of miR-4508-rich TDE-mediated induction and analysis of PMN formation and tumor metastasis in mice. (C)-(E) Expression of RFX1 (C), FN (D), and α-SMA (E) in the lungs of mice treated by miR-4508-rich TDEs as determined by IF staining. (F) IF staining of CD11b+Ly6G+ cells in the pre-metastatic lungs of mice. (G) *In vivo* live imaging of the metastatic tumors in mice preconditioned with miR-4508-rich exosomes (n=4). (H) Quantification of fluorescence in mice at indicated time points. (I) *Ex vivo* fluorescence images of the lungs with metastatic tumors. (J) and (K) HE (J) and IHC (K) staining of the metastatic tumors in lungs. *P < 0.05, **P < 0.01; each experiment was repeated at least 3 times; *P < 0.05, **P < 0.01.

We first determined whether miR-4508 inhibits RFX1 expression and activates murine fibroblasts WML2 (Fig. 5A). The biological effects of miR-4508 on PMN formation and tumor metastasis were studied *in vivo*. Exosomes were collected from Hepa1-6 cells infected with lentiviruses carrying miR-4508 or miR-NC. MiR-4508-enriched exosomes were injected into the tail vein of BALB/c mice. After three weeks, the lungs were excised in a subset of animals and subjected to IF staining for PMN analysis. The remaining mice were injected with H22-Luc cells (Fig. 5B). MiR-4508-enriched TDEs caused decreased RFX1 expression in the mouse lungs (Fig. 5C). Furthermore, miR-4508-enriched exosomes upregulated the expression of α-SMA and FN (Fig. 5D and 5E). The number of CD11b+Ly6G+ cells also increased following miR-4508-enriched exosome treatment (Fig. 5F). Lung metastasis occurred earlier in miR-4508-enriched exosome-treated mice than in miR-NC-treated mice (Fig. 5G and 5H). Bioluminescence imaging revealed an increase in distant metastatic tumors after three weeks in the isolated lungs of mice treated with miR-4508-enriched exosomes (Fig. 5I). H&E staining showed that the number and area of lung metastases in miR-4508-enriched exosome-treated mice were significantly increased compared to those in miR-NC-treated animals (Fig. 5J). IHC staining confirmed the high expression of Ki-67 in the lungs of miR-4508-enriched exosome-treated mice (Fig. 5K).

To specify that the pre-metastatic effect of miR-4508 was exerted by educated fibroblasts rather than tumor cells. We then investigate the direct effect of miR-4508 on tumor cell proliferation and invasion (Fig. S4A and S4B). Firstly, we found that miR-4508 mimic transfection and hypoxia treatment has no significant effect on proliferation ability of LM3 cells (Fig. S4B). Whereas, hypoxia significantly promotes migration of tumor cells while miR-4508 inhibitor has no significant effect on hypoxia-promoted tumor migration (Fig. S4A). Hence, we proposed that miR-4508, which is upregulated in TDE by hypoxia treatment, has no direct effect on proliferation and invasion of tumor cells.

### Downregulated RFX1 activates the p38 MAPK-NF-κB signaling pathway in fibroblasts

**Figure 6:**
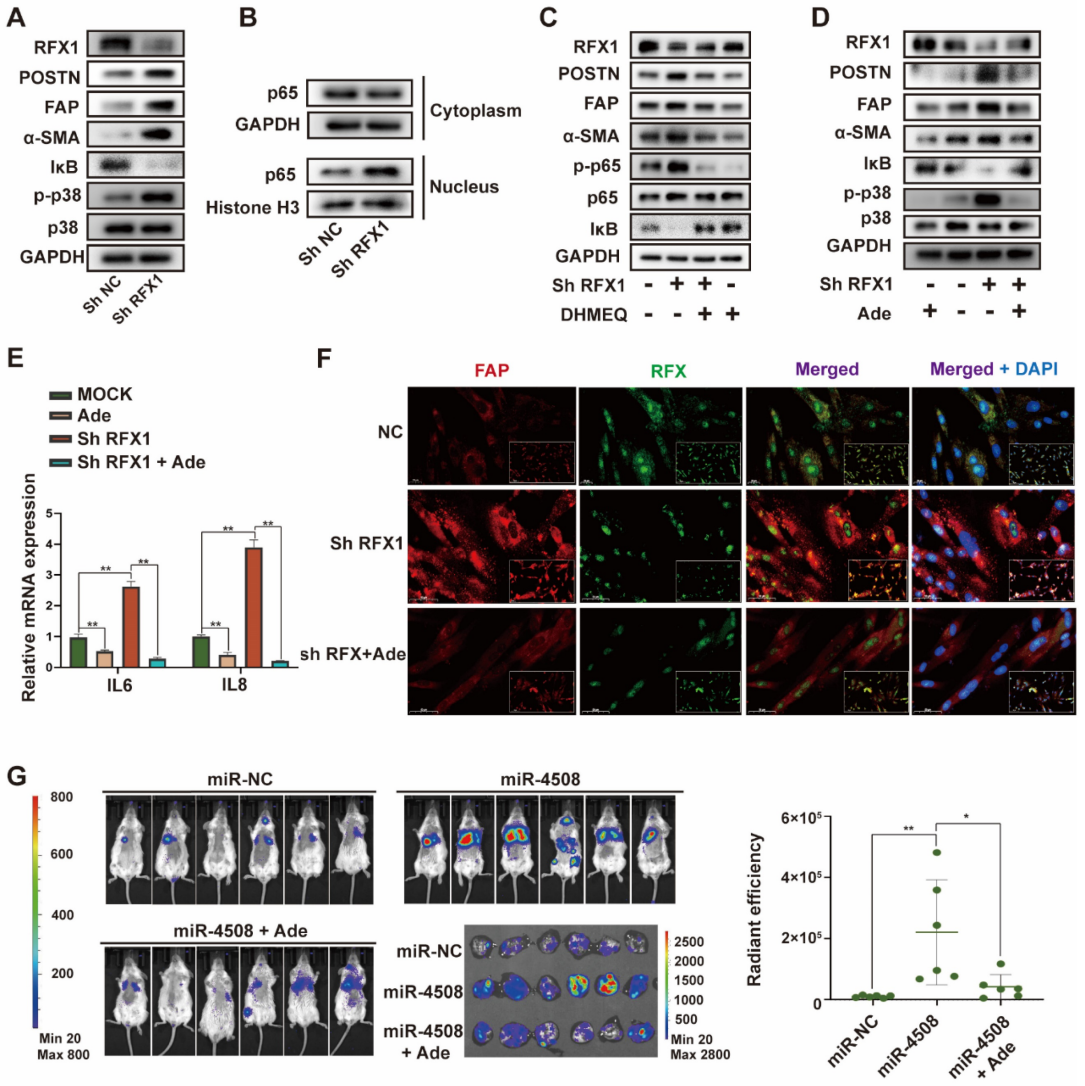
Downregulated RFX1 activates p38 MAPK-NF-κB in fibroblasts. (A) Effect of RFX1 knockdown on p38 MAPK activation and IκB degradation. (B) Levels of cytoplasmic and nuclear p65 in MRC5 cells transfected with RFX1 shRNA. (C) Effect of the NF-κB inhibitor DHMEQ on RFX1 knockdown-induced activation of fibroblasts and NF-κB. (D) Effect of the p38 inhibitor adezmapimod on RFX1 knockdown-induced activation of fibroblasts and p38 MAPK. (E) Effect of adezmapimod on RFX1 knockdown-induced upregulation of *IL6* and *IL8* mRNA. (F) IF staining of FAP and RFX1 in MRC5 cells treated with RFX1 shRNA with or without adezmapimod. (G) Effect of adezmapimod treatment on miR-4508-enriched exosome-induced metastatic tumor formation in mice. Each bar represents the mean ± S.D; each experiment was repeated at least 3 times; *P < 0.05, **P < 0.01.

Next, we explored the mechanisms underlying RFX1 decrease-induced fibroblast activation. GO analysis identified MAPKs as significant downstream signaling pathways of miR-4508 (Fig. S5). In particular, p38 MAPK plays an important role in fibroblast activation and PMN formation 29. We found that RFX1 knockdown increased p38 MAPK phosphorylation (Fig. 6A). NF-κB, a regulator of pro-inflammatory cytokine secretion, can also be triggered by p38 MAPK activation. Consistent with this, RFX1 knockdown led to IκB degradation (Fig. 6A). Moreover, the level of p65 in the cytoplasm was markedly downregulated whereas that in the nucleus was upregulated (Fig. 6B), implying translocation of the p65 subunit from the cytoplasm to the nucleus. Together, such IκB and NF-κB p65 regulation implied activation of the NF-κB signaling pathway.

We used the NF-κB inhibitor DHMEQ and p38 MAPK inhibitor adezmapimod to verify the roles of NF-κB and p38 MAPK in RFX1 knockdown-induced MRC5 cell activation. The decreased IκB and increased p-p65 levels caused by RFX1 knockdown were reversed by DHMEQ treatment (Fig. 6C). Adezmapimod reversed RFX knockdown-induced p38 MAPK phosphorylation and IκB inhibition (Fig. 6D). Moreover, pretreatment with either DHMEQ or adezmapimod blocked RFX1 knockdown-induced α-SMA, FAP, and POSTN upregulation (Fig. 6C and 6D), indicating that RFX1 knockdown induces fibroblast activation via p38 MAPK-NF-κB. In addition, adezmapimod prevented RFX1 shRNA-induced upregulation of *IL6* and *IL8* mRNA (Fig. 6E). IF staining also showed that adezmapimod neutralized the RFX1 knockdown-induced upregulation of FAP (Fig. 6F). In mice, adezmapimod blocked miR-4508-enriched exosome-induced lung metastasis (Fig. 6G). Taken together, the results indicate that RFX1 downregulation may activate the p38 MAPK-NF-κB signaling pathway to promote fibroblast activation.

### RFX1 activates p38 MAPK by upregulating IL17A

Although RFX1 downregulation evoked p38 MAPK activation in fibroblasts, an association between RFX1 and p38 MAPK has not previously been reported. We therefore further explored the means by which RFX1 mediates p38 MAPK activation in fibroblasts. Among previously reported RFX1 targets 30, IL17A was selected via functional analysis 31. IL17A is a pro-inflammatory cytokine produced by Th17 cells that can drive inflammatory pathology during infection and autoimmune diseases 32. As the IL17A receptor is expressed on fibroblasts 33, IL17A may stimulate fibroblasts to secrete pro-inflammatory cytokines, thereby supporting the growth and differentiation of immune cells 34. In addition, IL17A is associated with α-SMA expression in lung fibrotic foci 35. Therefore, we investigated the precise role of IL17A in RFX1 knockdown-induced fibroblast activation.

**Figure 7:**
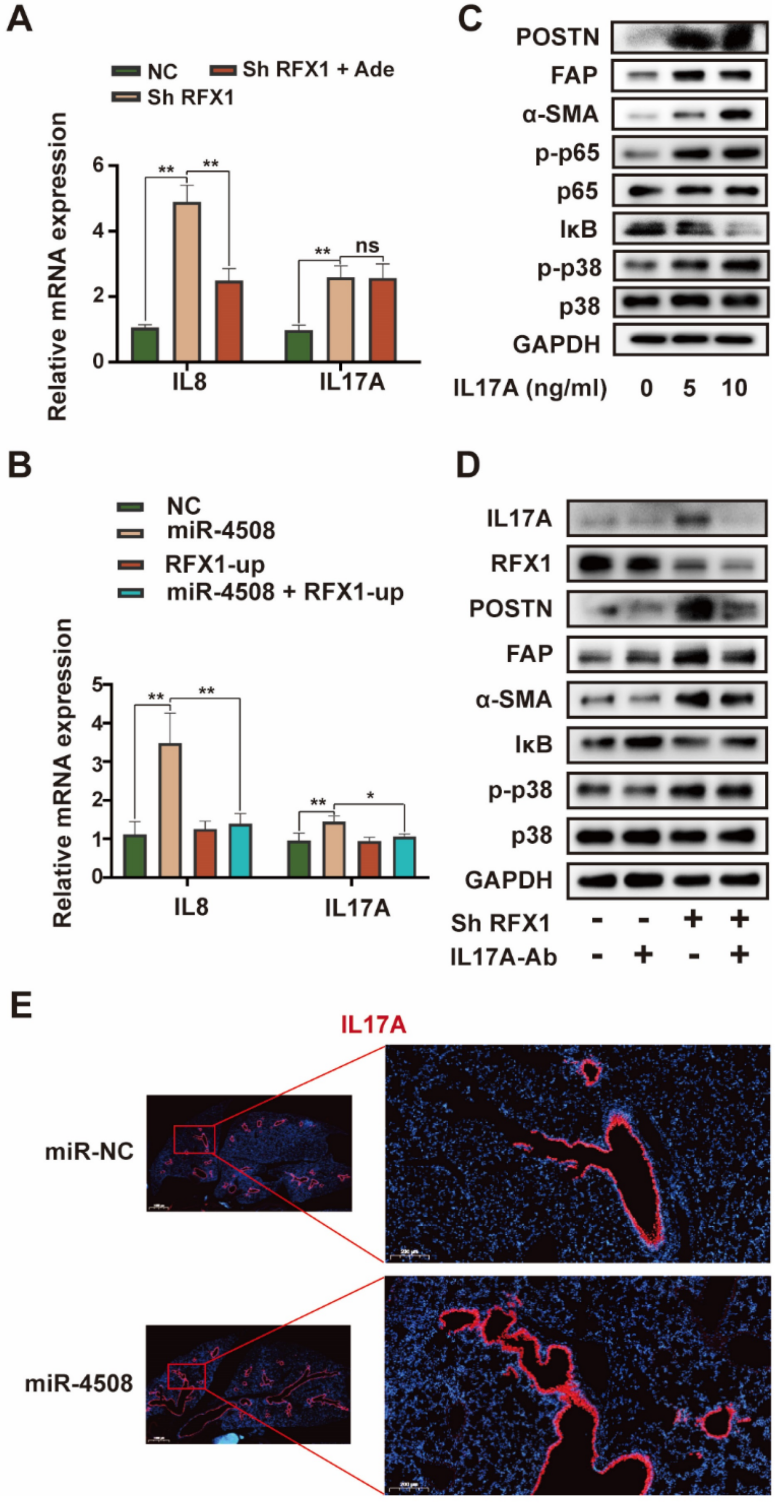
RFX1 activates p38 MAPK by upregulating IL17A. (A) Relative expression of* IL8* and *IL17A* mRNA in MRC5 cells treated with RFX1 shRNA with or without the p38 inhibitor adezmapimod. (B) Relative expression of *IL8* and* IL17A* mRNA in MRC5 cells transfected with miR-4508 mimics and/or an RFX1 overexpression plasmid. (C) Effect of IL17A on p38 MAPK-NFκB activation in MRC5 cells. (D) Effect of an IL17A neutralizing antibody on RFX1 knockdown-induced activation of fibroblasts and p38 MAPK. (E) IF staining of IL17A in the lungs of mice treated with miR-4508-rich exosomes. Each bar represents the mean ± S.D.; each experiment was repeated at least 3 times; *P < 0.05, **P < 0.01.

RFX1 knockdown significantly increased the mRNA expression of IL8 and IL17A in MRC5 cells (Fig. 7A). We also used adezmapimod to verify whether the increase in *IL8* and *IL17A* mRNA was dependent on p38 MAPK. Adezmapimod inhibited the upregulation of *IL8* but not *IL17A* mRNA induced by RFX1 knockdown (Fig. 7A). Moreover, miR-4508 mimics significantly elevated the mRNA levels of IL8 and IL17A in MRC5 cells, whereas RFX1 overexpression neutralized this effect (Fig. 7B). We then determined whether IL17A activates fibroblasts and p38 MAPK. As expected, IL17A stimulation activated fibroblasts and triggered p38 MAPK phosphorylation (Fig. 7C). In MRC5 cells, IL17A stimulation also upregulated phosphorylated p65 NF-κB and downregulated IκB levels (Fig. 7C). To confirm whether RFX1 knockdown-induced fibroblast activation was mediated by IL17A, an IL17A neutralizing antibody was used. RFX1 knockdown-induced upregulation of POSTN, FAP, and-SMA was blocked by an IL17A neutralizing antibody (Fig. 7D). RFX1 knockdown-induced p-38 MAPK phosphorylation and IκB depletion were also reversed (Fig. 7D). In mice, the expression of IL17A in the lungs was increased by treatment with miR-4508-enriched exosomes (Fig. 7E).

### *RXF1* expression negatively correlates with tumor lung metastasis

**Figure 8:**
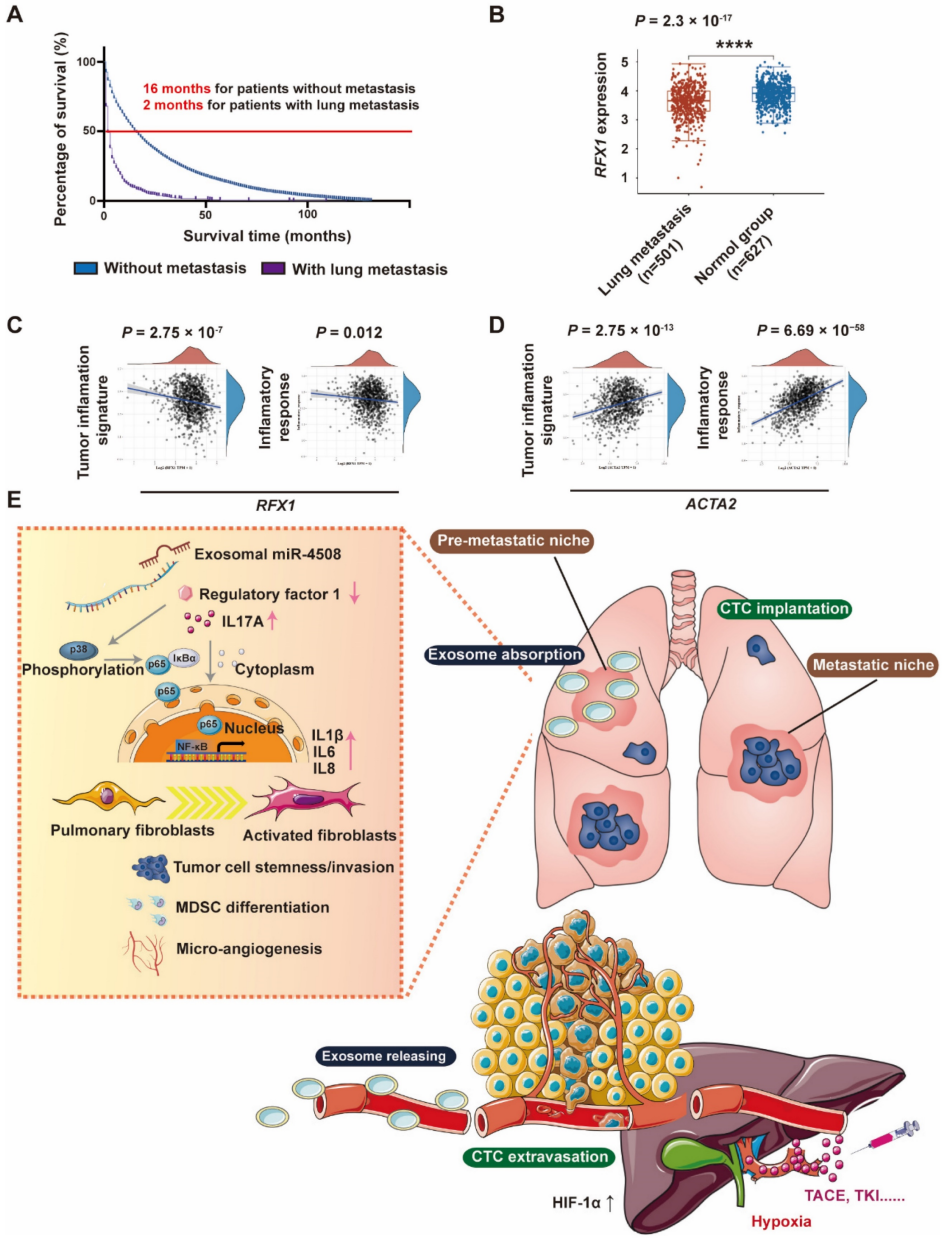
RFX1 negatively correlates with metastatic lung cancer. (A) Kaplan-Meier survival analysis based on data from Surveillance, Epidemiology, and End Results (SEER) database to investigate median survival time of patients with HCC with or without lung metastasis. (B) Data analysis of *RFX1* expression in patients with and without metastatic lung cancer from TCGE database. (C) and (D) PCA analysis of the correlation between *RFX1* (C) and *ACTA2* (D) expression and tumor signature score. (E) Proposed mechanism of H-TDE-induced lung PMN formation.

Through Kaplan-Meier survival analysis based on data from Surveillance, Epidemiology, and End Results (SEER) database (https://seer.cancer.gov/), we discovered that patients with HCC in presence of lung metastasis have worse median survival time (2 months) compared to those without metastasis (16 months) (Fig. 8A). To further analyze the clinical correlation between *RFX1* expression and the development of pulmonary metastases, RNA-sequencing profiles and clinical information for lung metastasis and normal tissues were downloaded from TCGA database. Pulmonary *RFX1* expression was significantly down-regulated in patients with metastatic lung cancer (Fig. 8B). To further explore the relationship between genes and inflammation-related pathways, principal component analysis was applied to the log2-transformed TPM values of *RFX1* and *ACTA2* (the gene encoding α-SMA). Correlations between individual genes and pathway scores, including the inflammatory response and tumor inflammation signature, were analyzed using the Spearman correlation. The pulmonary *RFX1* level negatively correlated with each of the two inflammatory signatures, whereas ACTA2 positively correlated with tumor inflammation (Fig. 8C and 8D).

## Discussion

TACE is recommended as the first-line treatment for patients with intermediate stage HCC 36. Although local tumors generally benefit from TACE, subsequent intra-arterial embolization aggravates local ischemia and hypoxia 13,14. The hypoxic microenvironment promotes tumor progression and metastasis; however, to date, few studies have investigated the mechanisms underlying distant metastasis of HCC caused by TACE-mediated regional hypoxic microenvironments. Therefore, it is necessary to investigate how hypoxia promotes the progression of local residual tumors. In this study, we determined the effects of H-TDEs on fibroblast activation* in vitro*. We then analyzed the differences in exosomal miRNA profiles between hypoxic and normoxic tumor cells. The functions of the differentially expressed miRNAs were examined and miR-4508 was identified as the strongest activator of fibroblasts transferred by H-TDEs. MiR-4508 activated fibroblasts to form PMNs in the lung and promoted metastatic tumor growth by depleting its target, RFX1, to trigger the IL17A-p38 MAPK-NF-κB signaling pathway. Furthermore, our findings indicated that plasma exosomes of patients with HCC underwent TACE harbored high miR-4508 load. The results of this study elucidated the communication between hypoxic HCC cells and lung fibroblasts, together with the underlying molecular mechanism of hypoxia-induced lung metastasis in HCC.

In the lungs, the activation of quiescent resident fibroblasts into pro-inflammatory cells is a critical link in pulmonary PMN formation, accompanied by ECM remodeling, increased vascular leakiness, inflammatory factor release, and immunosuppressive cell recruitment 37,38. Prevention of fibroblast activation impedes PMN formation 39. Exosomes mediate communication between host and recipient cells 40. TDEs stably exist in the blood circulation and contribute to the formation of PMNs in distant organs by transferring multiple cargoes 41,42. For example, exosomal miR-1247-3p secreted by high-metastatic HCC converts fibroblasts to cancer-associated fibroblasts (CAFs) to form PMNs by directly targeting B4GALT3 and activating β1-integrin-NF-κB signaling 43. In colorectal cancer, exosomal HSPC111 facilitates PMN formation by regulating the lipid metabolism of CAFs through the phosphorylation of ATP-citrate lyase 44.

Hypoxia largely influences the interaction between tumor cells and the local microenvironment, and plays an important role in tumor metastasis. Hypoxia may enhance the secretion of exosomes by cancer cells and alter miRNA signatures in several tumor types, including HCC 45-48. Hypoxia triggers colorectal cancer to release exosomes containing high levels of miR-135a-5p, which can be phagocytosed by Kupffer cells from the blood circulation into the liver 45. In pancreatic stellate cells, hypoxia elevates miR-4465 and miR-616-3p expression in TDEs, and the transfer of these miRNAs promotes the progression and metastasis of pancreatic cancer by suppressing the PTEN/AKT pathway 47. In HCC, hypoxia increases miR-1273f expression within Huh7-derived exosomes; notably, miR-1273f enhances the malignant phenotypes of normal HCC cells through the Wnt/β-catenin signaling pathway 46.

In the present study, we discovered that H-TDEs upregulated the expression of FAP, FN, and α-SMA in the fibroblasts and lungs of mice, indicative of the activation of pulmonary fibroblasts, leading to increased secretion of pro-inflammatory cytokines, recruitment of MDSCs, and ultimately increased pulmonary metastatic tumors. We also showed that plasma-derived exosomes from patients with HCC following TACE could activate fibroblasts, suggesting that hypoxia-induced exosomes from HCC cells promote PMN formation in the lungs and accelerate pulmonary metastasis. In both H-TDEs and plasma-derived exosomes, miR-4508 overexpression activated fibroblasts and promoted PMN formation in the lungs.

However, the role of miR-4508 in malignant tumors has not yet been clearly elucidated. Bian *et al*. 49 showed that miR-4508 expression is decreased in tumor tissues and breast cancer cells and negatively correlates with that of Ki-67. Boo *et al.* 50 found that miR-4508 is upregulated in spheroid MCF-7 cells compared with the level in parental cells and exhibits a potential association with chemoresistance and self-renewal capability. In colorectal cancer, STAT3 siRNA significantly decreases miR-4508 levels 51. Chu *et al*. 52 identified miRNA-4508 in peripheral blood lymphocytes as a potential diagnostic biomarker of silica-related pulmonary fibrosis. Nevertheless, the mechanisms underlying tumor-regulating activity of miR-4508 remain to be elucidated.

In this study, RFX1, a context-dependent transcription factor, was identified as a target of miR-4508. The RFX1 protein contains an evolutionarily conserved winged-helix-type DNA-binding domain and has been identified as a critical DNA-binding factor that targets the X-box sequence in MHC class II genes 11. RFX1 is a central link in the regulation of cancer-related gene networks 30. In human glioblastoma cells, RFX1 downregulates fibroblast growth factor 1 expression 53. Additionally, RFX1 overexpression may reduce the proliferation and invasion of glioblastoma cells 54. In patients with HCC, downregulated RFX1 is correlated with poor prognosis and high recurrence risk, whereas RFX1 overexpression decreases cancer invasion and epithelial-mesenchymal transition 55, which plays a crucial role in the early stages of metastasis 56,57. Moreover, RFX1/SHP1 activation overcomes STAT3-mediated sorafenib resistance in HCC 58. However, the role of RFX1 in fibroblasts has not previously been investigated.

We found that downregulation of RFX1 activated fibroblasts. Pro-inflammatory genes are targets of p38 MAPK and NF-κB, which have been demonstrated to be involved in fibroblast activation. Although RFX1 knockdown activated p38 MAPK and NF-κB signaling in fibroblasts, the links between RFX1 and p38 MAPK/NF-κB have not previously been established. RFX1 not only targets oncogenes (such as *c-MYC*) but also downregulates some inflammation-related genes with pro-tumor activities (such as *TGF-β2*, *IL17A*, and *TLR4*) 27. Numerous studies have indicated that IL17A promotes the release of inflammatory factors and contributes to the development of pulmonary fibrosis 59,60. Our results showed that RFX1 deficiency resulted in IL17A overexpression in MRC5 cells and the lungs of mice. RFX1 deficiency leads to IL17A overexpression through increased histone H3 acetylation and decreased H3K9 tri-methylation 54. Moreover, IL17A shapes the transcriptional program of fibroblasts toward a protumorigenic function 61. We discovered that IL17A is involved downstream of RFX1 deficiency and stimulates the p38 MAPK signaling pathway in TDE-related fibroblast activation (Fig. 8C). Our study elucidates a new mechanism for the communication between HCC cells in a hypoxic microenvironment and lung fibroblasts to trigger PMN formation and promote lung metastasis. These results may also provide a therapeutic target to prevent metastasis of HCC, especially in patients treated with TACE.

## Conclusion

In conclusion, our study demonstrated that the hypoxic microenvironment in HCC boosted the release of exosomes containing high levels of miR-4508, which promoted pulmonary PMN development by decreasing RFX1, thereby activating the IL17A-p38 MAPK-NFκB signaling pathway in fibroblasts. Moreover, TACE also led to the overexpression of miR-4508 in the plasma exosomes of patients with HCC.

## Supplementary Material

Supplementary figures.
