# Supplementary material for: Hypoxia-induced exosomes facilitate lung pre-metastatic niche formation in hepatocellular carcinoma through the miR-4508-RFX1-IL17A-p38 MAPK-NF-κB pathway
Source: Int J Biol Sci. 2023 Sep 4;19(15):4744–62. doi: 10.7150/ijbs.86767 (PMC10539707; doi:10.7150/ijbs.86767)
Supplement: Supplementary file 1 — Supplementary figures. [file ijbsv19p4744s1.pdf]

Supplementary figures

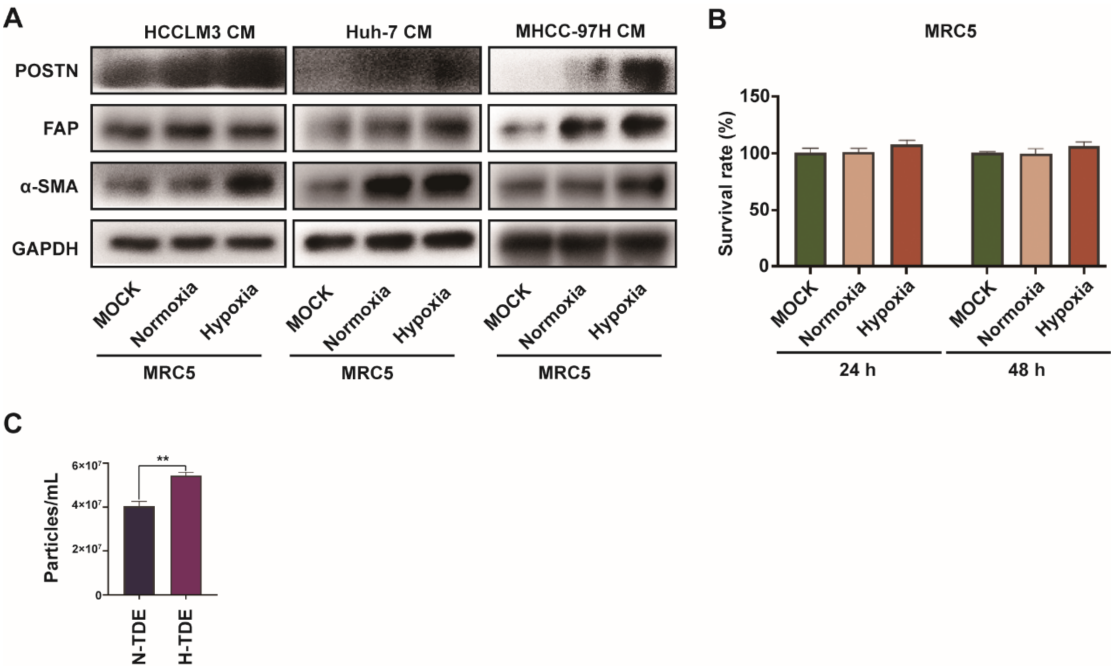

Figure S1.

A: Fibroblast activation markers expression in MRC5 treated by tumor cell-derived conditioned medium. B: The survival rate of MRC5 treated by tumor cell-derived conditioned medium determined by CCK-8. C: the quantity of TDE secreted by equal quantity of LM3 cells were detected by nanoparticle tracking analysis.

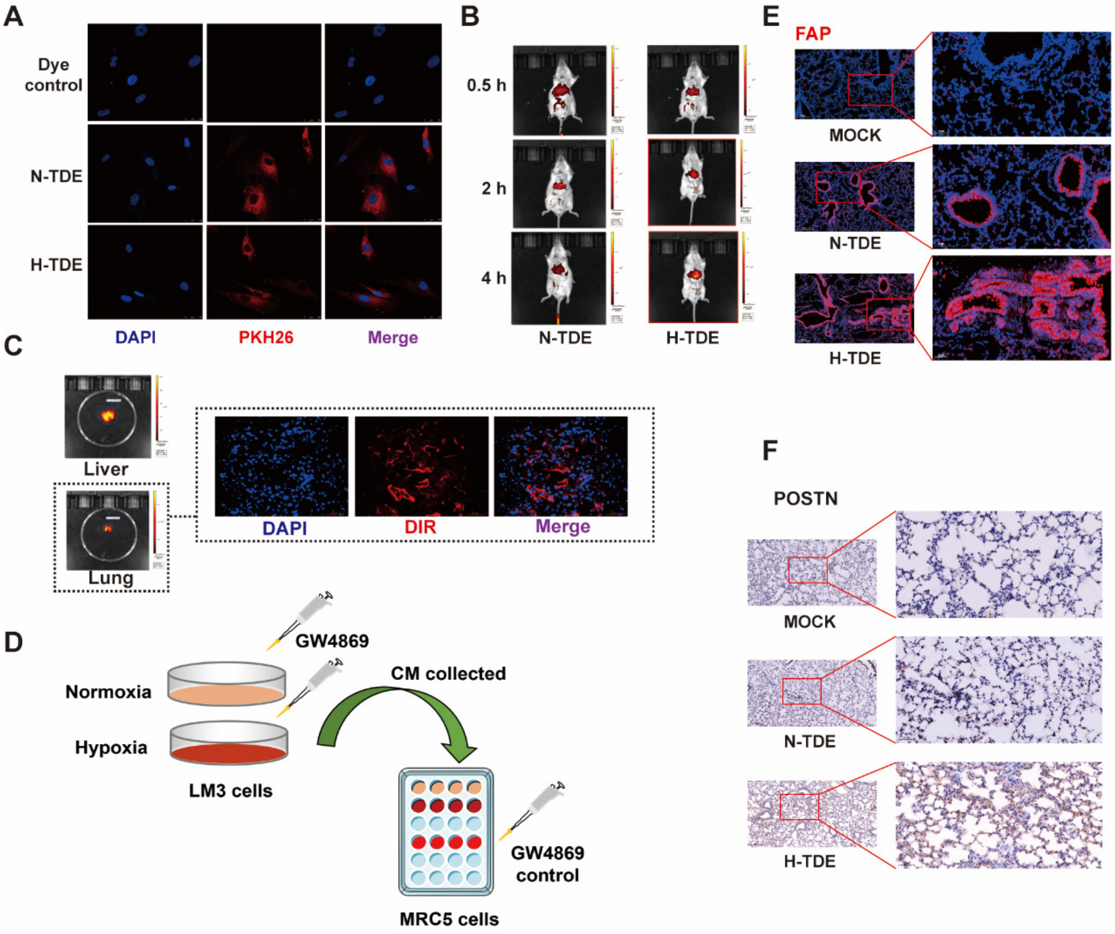

Figure S2.

A: PKH26-labelled tumor-derived exosomes were co-incubated with MRC5 cells. The fluorescent photograph showed the exosomes intake ability of MRC5 cells. B. The in vivo distribution of DIR-labelled tumor-derived exosomes at specific time points after injection determined by living imaging system. C: Images taken from the frozen section under fluorescence microscope. D: Schematic diagram of GW4869-mediated exosome blocking test. E: Immunofluorescence staining of FAP in the lungs of mice treated with TDE. F: Immunohistochemical staining of POSTN in the lungs of mice treated with TDE.

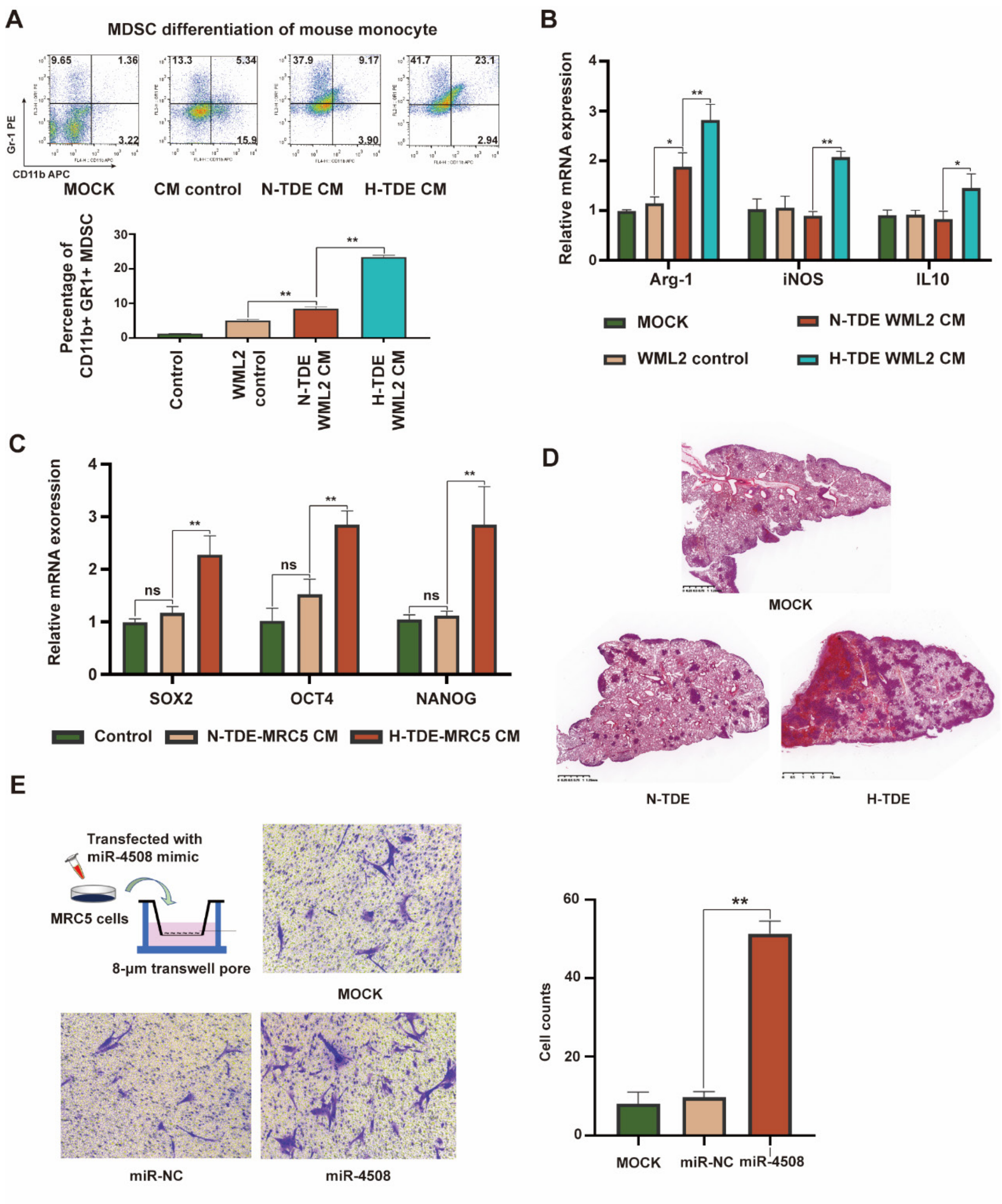

Figure S3.

A: MDSC differentiation of mice monocytes cultured in TDE-treated WML2 conditioned medium was detected by flow cytometry. B: The level of immunosuppressive factors expressed in mice monocytes which were cultured in TDE-treated WML2 conditioned medium was determined by qRT-PCR. C: The tumor stemness markers including SOX2, OCT4 and NANOG expressed in LM3 tumor cells

treated with conditioned medium from educated MRC5 cells. D: Representative panoramic paragraph of H&E-stained slides from pulmonary tissue of mice treated with TDEs and injected i.v. with H22 tumor cells. E: The effect of miR-4508 on the migration ability of MRC5 cells determined by transwell assay. **P<0.01.

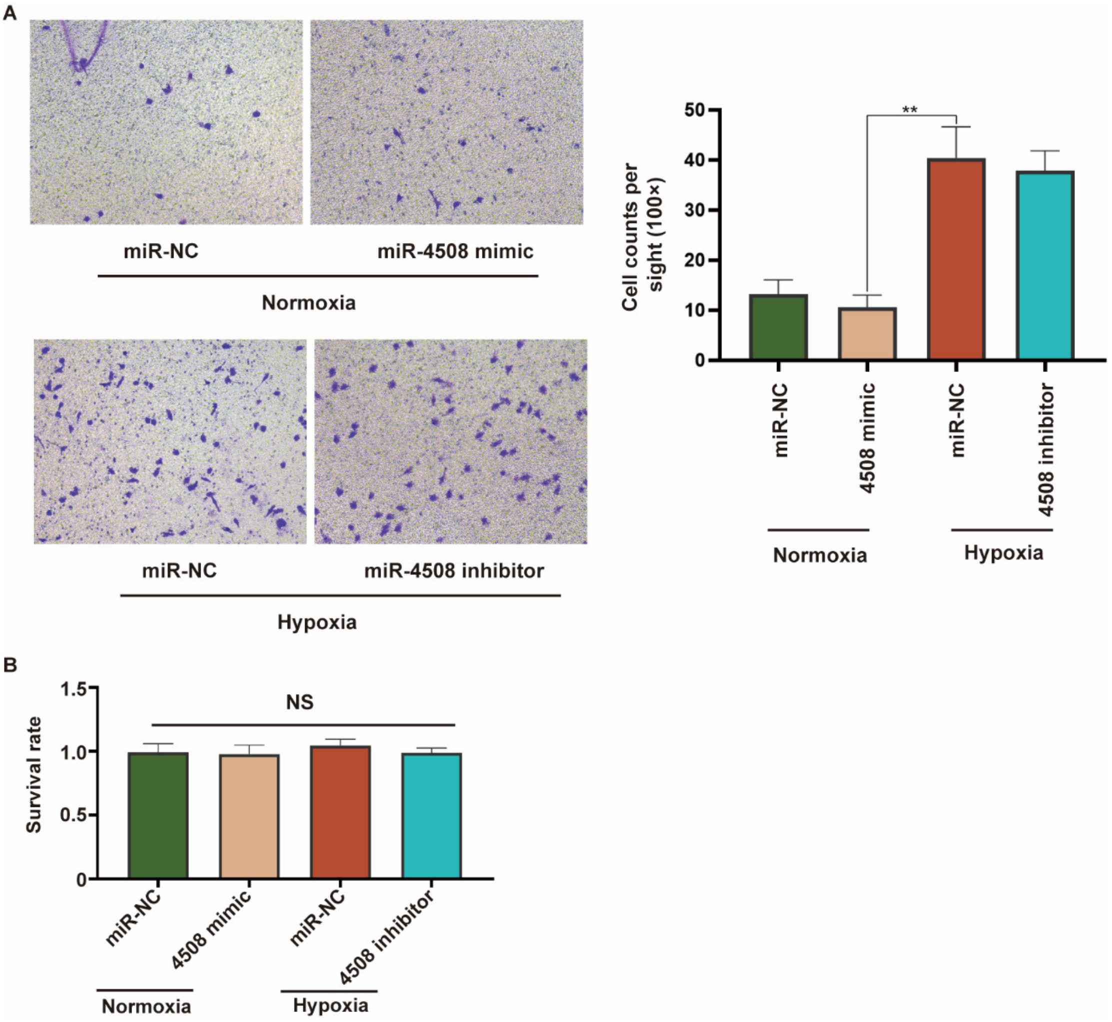

Fig. S4.

A: The effect of miR-4508 and hypoxia environment on the migration ability of LM3 tumor cells determined by transwell assay. B: The effect of miR-4508 and hypoxia environment on the proliferation ability of LM3 tumor cells determined by CCK8 assay. **P<0.01.

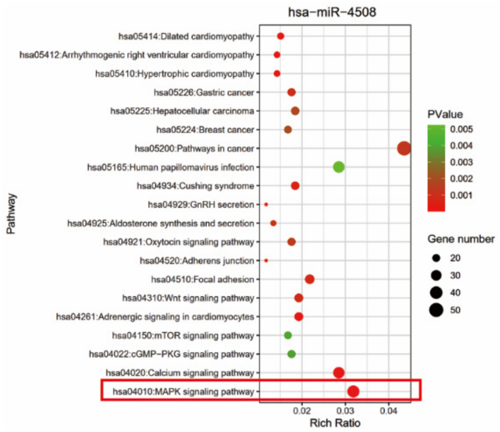

Figure S5.

Pathway analysis of downstream signals of miR-4508.
